# An investigation of a genomewide supported psychosis variant in *ZNF804A* and white matter integrity in the human brain^☆^

**DOI:** 10.1016/j.mri.2012.05.013

**Published:** 2012-12

**Authors:** Emma Sprooten, Andrew M. McIntosh, Stephen M. Lawrie, Jeremy Hall, Jess E. Sussmann, Norbert Dahmen, Andreas Konrad, Mark E. Bastin, Georg Winterer

**Affiliations:** a Division of Psychiatry, University of Edinburgh, EH10 5HF, United Kingdom; b Department of Psychiatry and Psychotherapy, University Medical Center Mainz, 55131, Germany; c Division of Health Sciences (Medical Physics), Western General Hospital, University of Edinburgh, EH4 2XU, United Kingdom; d Institute of Medicine and Neurosciences, Helmholtz Research Center Jülich, 52425, Germany; e Cologne Center for Genomics, University of Cologne, 50931, Germany

**Keywords:** *ZNF804A*, White matter, Structural connectivity, Diffusion tensor MRI, Intermediate phenotype, Genetic risk

## Abstract

*ZNF804A,* a genomewide supported susceptibility gene for schizophrenia and bipolar disorder, has been associated with task-independent functional connectivity between the left and right dorsolateral prefrontal cortices. Several lines of evidence have converged on the hypothesis that this effect may be mediated by structural connectivity. We tested this hypothesis using diffusion tensor magnetic resonance imaging in three samples: one German sample of 50 healthy individuals, one Scottish sample of 83 healthy individuals and one Scottish sample of 84 unaffected relatives of bipolar patients. Voxel-based analysis and tract-based spatial statistics did not detect any fractional anisotropy (FA) differences between minor allele carriers and individuals homozygous for the major allele at rs1344706. Similarly, region-of-interest analyses and quantitative tractography of the genu of the corpus callosum revealed no significant FA differences between the genotype groups. Examination of effect sizes and confidence intervals indicated that this negative finding is very unlikely to be due to a lack of statistical power. In summary, despite using various analysis techniques in three different samples, our results were strikingly and consistently negative. These data therefore suggest that it is unlikely that the effects of genetic variation at rs1344706 on functional connectivity are mediated by structural integrity differences in large, long-range white matter fiber connections.

## 1 Introduction

In the first large genomewide association study of schizophrenia, the common single nucleotide polymorphism (SNP) rs1344706 of the Zinc Finger Protein 804A gene (*ZNF804A*) was identified as the most significant genetic marker (*P*< 1.61×10^− 7^) [1]. Combining schizophrenia and bipolar phenotypes showed an even higher association (*P*< 9.96×10^− 9^), surpassing genomewide significance at *P*< 7.2×10^− 8^. Four independent replications have since confirmed its association with schizophrenia and bipolar disorder [2–4], and a meta-analysis resulted in *P* values up to 4.1×10^− 13^ for the combined phenotype [5].

Despite this abundance of statistical evidence for an association of *ZNF804A* with psychosis, only modest effect sizes have been reported with odds ratios of around 1.10 (95% confidence interval 1.07–1.14), and its functional mechanisms are unclear [6]. Intermediate phenotypes are therefore especially valuable, giving rise to larger expected effect sizes and requiring smaller sample sizes [7]. Two important prerequisites for intermediate phenotypes are that they are heritable and expressed in unaffected relatives of the affected patients. Substantial heritability of white matter integrity as measured with diffusion tensor magnetic resonance imaging (DT-MRI), and in particular of fractional anisotropy (FA), has been firmly established, with heritability estimates (*h*^2^) ranging between 0.4 and 0.8 depending on brain structure, for example, the genu of corpus callosum with *h*^2^ estimated at 0.66 [8,9]. There is also growing evidence of abnormal white matter integrity in psychosis as measured by DT-MRI [10,11] and in association with genetic risk for schizophrenia and bipolar disorder [12–15]. Abnormalities in frontotemporal functional connectivity are also found in siblings of patients with schizophrenia [16,17], but the heritability of functional connectivity determined from functional MRI (fMRI) is less well established, with one study estimating *h*^2^ at 0.42 [18].

It is known that *ZNF804A* is highly expressed in the brain and that the presence of A-allele at rs1344704 creates a myelin transcription factor binding site [2,19]. The most comprehensive data on *ZNF804A* function come from neuroimaging and neuropsychology, collectively indicating that rs1344706 is associated with brain function. Esslinger et al. [20] reported reduced functional connectivity between the left and right dorsolateral prefrontal cortices and increased frontotemporal functional connectivity in carriers of the risk allele (A) during a working memory task, findings that were (partly) replicated in two subsequent studies [16,21]. Importantly, Esslinger et al. [22] later showed that the reduced interhemispheric prefrontal connectivity was also apparent during a facial emotion processing task and during rest, whereas the increased frontotemporal connectivity appeared specific to working memory processes both in the original study and in two replication studies [16,21]. This task-independent association of *ZNF804A* genotype on interhemispheric prefrontal functional connectivity prompted the hypothesis that these effects may be mediated by effects on white matter integrity, especially in anterior interhemispheric connections. In contrast, the effects of *ZNF804A* on frontotemporal connectivity are less likely to be directly mediated by white matter structure since they have only been observed in the context of working memory tasks [21,22] and interact with task condition [16].

In line with this hypothesis, Lencz et al. [23] showed that individuals homozygous for the *ZNF804A* risk allele (A) have reduced total white matter volumes compared to carriers of the nonrisk allele (C). However, total volumetric measures lack spatial specificity and are particularly susceptible to partial volume effects and segmentation difficulties. DT-MRI is more suited to the study of white matter, and FA is the most commonly used measure of white matter integrity in vivo. Surprisingly, using DT-MRI tractography, Voineskos et al. [19] did not detect any effects of *ZNF804A* on FA in the uncinate fasciculi, arcuate fasciculi, cingulum or corpus callosum of 62 healthy individuals, 39 C-carriers versus 23 A-homozygotes, aged between 18 and 59 years.

Taken together, the myelin transcription factor binding site determined by the A-allele at rs1344706, the task-independent effects of *ZNF804A* on functional connectivity, the high heritability of FA and its association with genetic risk all converge in the hypothesis that structural connectivity is a mediator of rs1344706 effects on functional connectivity. This hypothesis is logically appealing and readily testable with FA as an objectively measurable approximation of white matter integrity. In the present study, we investigated possible effects of *ZNF804A* on FA using whole-brain voxel-based analysis and tract-based spatial statistics (TBSS). Since the only connection affected by *ZNF804A* independent of task was between the left and right prefrontal cortices [22], we further investigated the anterior part of the corpus callosum as a particular region of interest (ROI) using quantitative tractography and atlas-based ROI analyses. Because proving equivalence statistically entails more than the absence of significant difference, any negative findings were corroborated with an extensive examination of statistical power and effect sizes.

## 2 Materials and methods

DT-MRI and genotype data were analyzed separately in three samples: a German sample consisting of 50 healthy individuals, a Scottish sample of 83 healthy controls and a Scottish sample of 84 unaffected relatives of patients with bipolar disorder.

### 2.1 Germany

#### 2.1.1 Subjects

Fifty-nine healthy young Caucasian subjects (mean age: 22.7±1.7 years, range: 18–26 years, 27 males) were investigated. Participants were only included if there was no evidence for any medical or neurological condition that could interfere with the purpose of the study and if there was no history of any psychiatric *Diagnostic and Statistical Manual of Mental Disorders, Fourth Edition* (DSM-IV) axis I or axis II disorder including current or recent drug or alcohol abuse as assessed by a structured clinical interview [24]. A formal medical and neurological examination, including urine toxicology for illegal drug abuse screening, routine blood tests and a clinical electroencephalographic session, was also performed. The subjects did not have a family history of schizophrenia or bipolar disorder, and all were right-handed. IQ was assessed with the HAWIE-R (Hamburg-Wechsler Intelligenztest) Scale [25], which is largely equivalent to the full-scale Wechsler Adult Intelligence Scale-R [26].

#### 2.1.2 Genotyping

DNA was obtained from venous blood using standard techniques. SNP rs1344706 from the *ZNF804A* gene was genotyped by the analysis of primer extension products generated from amplified genomic DNA using a Sequenom (Sequenom Inc., San Diego, CA, USA) chip-based Matrix-assisted laser desorption/ionization Time-of-Flight (MALDI-TOF) mass spectrometry platform. In brief, polymerase chain reaction (PCR) and extension reactions were designed using MassARRAY design software (Sequenom Inc.) and were carried out using 2.5 ng of template DNA. Unincorporated nucleotides in the PCR product were deactivated using shrimp alkaline phosphatase. The primer extension products were then cleaned and spotted onto a SpectroChip with a massARRAY nanodispenser. The chips were scanned using a mass spectrometry workstation (MassARRAY compact analyzer, Sequenom Inc.), and the resulting spectra were analyzed and genotypes determined using the Sequenom SpectroTYPER-RT software with a call rate of 100%.

#### 2.1.3 Structural and DT-MRI data acquisition

MRI scanning was performed using a Siemens Sonata 1.5-T clinical system (Siemens Healthcare, Erlangen, Germany). High-resolution T1-weighted MRI volume scans were acquired using a magnetization prepared rapid gradient echo sequence with 176 contiguous slices of 1-mm thickness, field-of-view 256×256 mm, acquisition matrix 256×256, flip angle 15°, repetition time (TR) 2860 ms and echo time (TE) 3.9 ms. DT-MRI was performed using a single-shot spin-echo echo-planar imaging (EPI) sequence (TR 8000, TE 100 ms) with diffusion encoding gradients applied in six noncollinear directions (*b*= 1000 s/mm²) and one acquisition without diffusion encoding (*b*= 0 s/mm²). A generalized autocalibrating partially parallel acquisition reconstruction algorithm was used. The acquisition matrix was 128×128 with a field of view of 192×192 mm and slice thickness of 2 mm, giving a voxel resolution of 1.5×1.5×2.0 mm³. Sixty-four axial slices were acquired to cover the whole brain without interslice gap. A total of 10 acquisitions were performed and averaged.

#### 2.1.4 Voxel-based morphometry and diffusion tensor analyses

Voxel-based morphometry (VBM) was carried out with an optimized VBM protocol [27] using SPM5 software (Statistical Parametric Mapping, Wellcome Department of Cognitive Neurology, London, UK) implemented in Matlab 7.1 (Mathworks Inc., Sherborn, MA, USA). The high-resolution T1-weighted MRI scans were normalized to a standard template and segmented into gray matter, white matter and cerebrospinal fluid. The segmented volumes were then smoothed with a 6-mm isotropic full-width-half-maximum (FWHM) Gaussian kernel.

FA and mean diffusivity (MD) were calculated for each voxel using the FDT toolbox of the FSL software library (FMRIB, Oxford, UK; http://www.fmrib.ox.ac.uk/fsl). The images were checked by eye for motion and other scanner artifacts, which led to the exclusion of nine participants. The T2-weighted volumes were then normalized to the Montreal Neurological Institute (MNI) T2-weighted template using SPM2 software implemented in Matlab 6.5. Identical normalization parameters were used for warping of the FA and MD volumes to standard MNI space. The resulting FA and MD volumes were then smoothed with a 6×6×6-mm FWHM Gaussian kernel to improve signal-to-noise ratio and normalization.

To compare subjects homozygous for the A-risk allele to C-carriers, voxel-wise *t* tests were performed in SPM on the normalized and smoothed T1-weighted, FA and MD volumes. We adopted a statistical threshold of *P*<.05, with false detection rate correction (FDR) for multiple comparisons. Moreover, to avoid false-negative findings, a second analysis was performed with an uncorrected threshold (*P*<.001), for which subthreshold cluster sizes were statistically examined using a nonstationary cluster inference toolbox for SPM5 based on random field theory [28].

### 2.2 Scotland

#### 2.2.1 Subjects

Participants were recruited as part of a large family study of bipolar disorder, as described in more detail elsewhere [15]. Individuals in the “high-risk” group had at least one first-degree or two second-degree relatives with a diagnosis of bipolar I disorder, confirmed using the Structured Clinical Interview for DSM-IV or the Operational Criteria Checklist (OPCRIT) [29]. Exclusion criteria were any axis 1 psychiatric disorder including substance dependence, major neurological disorders, history of head injury, history of learning disability or any contraindications to MRI examination. IQ was measured using the Wechsler Abbreviated Scale of Intelligence. In total, 115 high-risk subjects and 86 controls provided both DT-MRI data and blood samples for genotyping. Because some high-risk subjects were genetically related, only one of each family was randomly included to avoid statistical dependence in the sample, leaving 89 high-risk and 86 controls.

#### 2.2.2 Genotyping

DNA was isolated from venous blood samples, and genotypes at rs1344706 were determined using TaqMan polymerase chain reaction (PCR, TaqMan, AssayByDesign, Applied Biosystems, Foster City, CA, USA) using validated assays. Call rates were 0.95 for the control group and 0.96 for the high-risk group. The numbers of subjects in each genotype group did not deviate from the Hardy–Weinberg equilibrium for either sample (both *P*>.84).

#### 2.2.3 DT-MRI acquisition and preprocessing

Details about acquisition of DT-MRI data and preprocessing are available elsewhere [15]. Briefly, MRI data were collected using a GE Signa Horizon HDX 1.5-T clinical scanner (General Electric, Milwaukee, WI, USA). EPI diffusion weighted volumes (*b*= 1000 s/mm^2^) were acquired in 64 noncollinear directions along with seven T_2_-weighted scans. Fifty-three 2.5-mm contiguous axial slices were acquired, with field of view 240×240 mm and matrix 96×96, resulting in an isotropic voxel dimension of 2.5 mm. The data were corrected for eddy-current-induced distortions and bulk subject motion, the brain was extracted, and diffusion tensor characteristics including FA were calculated using standard software tools available from FSL. The resulting FA volumes were visually inspected, and three control participants (1CC, 1AA, 1AC) and five high-risk participants (2AA, 3AC) were excluded from further analyses due to motion or other scanner artifacts. The final Scottish sample included 84 high-risk and 83 control participants.

#### 2.2.4 TBSS and voxel-wise statistics

Voxel-based analysis of normalized and smoothed FA volumes is a practical and widely used technique for voxel-wise comparisons between subjects, with the advantage that all white matter is analyzed without the need for a priori ROI. However, given that white matter morphology varies between subjects and white mater structure can be very thin or individually shaped in places, voxel-based methods can be sensitive to partial volume and misregistration artifacts. TBSS is a method especially designed to investigate white matter structure and partially alleviates these potential biases [30,31]. In this method, all subjects' FA volumes are nonlinearly registered to a white matter “skeleton” template, and statistics computed for FA on a voxel-by-voxel basis within this common skeleton. Firstly, a white matter skeleton template is created based on the average FA volumes of the sample. Next, for each subject, the TBSS algorithm searches each voxel on the individual skeleton for the one with the highest FA nearby the skeleton template. This maximum voxel is then projected onto the common skeleton template, thus creating one skeleton for each subject, which is assumed to contain the centers of the white matter tracts that are common to all subjects. Voxel-wise statistics are then performed on these individual skeletons.

TBSS was carried out using standard procedures freely available from FSL [31]. The alignment of the skeleton template with each subject's FA volume was visually checked, and the template was thresholded at FA>0.2. (The TBSS skeleton template is shown in Supplementary Figure 1.) FA values within the skeletons were then compared between C-carriers and individuals homozygous for the A-allele in the control and high-risk groups separately using voxel-wise nonparametric *t* tests calculated by “randomise” in FSL. Statistics were corrected for multiple comparisons according to family-wise error (*P*<.05) using threshold-free cluster enhancement (TFCE) [32]. Additionally, to look for any clusters on trend level, the raw *T*-statistic images were thresholded at *T*> 3.41, equivalent to *P*<.001 (df=82). For the control group, an additional analysis was performed with age included as a covariate.

#### 2.2.5 Small volume correction (SVC) — ROI analysis

Because two previous studies [20,22] showed that *ZNF804A* was related to task-independent functional connectivity between the dorsolateral prefrontal cortices, an SVC was applied to include only voxels within the skeleton and the body and genu of corpus callosum (Supplementary Figure 2). In the presence of a priori hypotheses, the use of an SVC increases statistical power by restricting the analysis to a specific region, thereby decreasing the penalty of multiple comparison correction over many voxels.

The SVC was created using the John Hopkins University white matter labels atlas in MNI space [33], thresholded to include only the genu and body of corpus callosum, smoothed (FWHM 1.1 mm), binarized and multiplied with the skeleton mask to include only voxels that were in both the skeleton and the corpus callosum SVC. Voxel-wise analysis was rerun with this SVC applied as a mask.

In addition, using the SVC as an ROI, the average FA was extracted from this region and compared between genotype groups using independent-sample *t* tests. (Of note, by merging the body and genu of corpus callosum, we are compromising the power to detect any very focal signals, which are more likely to be detected in the voxel-wise analysis with SVC, while gaining power to detect more diffuse signals within the corpus callosum.) Because one extreme outlier (> 3 interquartile ranges from the median) was observed in the high-risk group, an additional analysis excluding this individual was performed.

#### 2.2.6 Quantitative tractography

ROIs like those described above require normalization, which again makes them susceptible to misregistration and partial volume effects. Tractography refers to the segmentation, or tracing, of major white matter fiber pathways in individual brains based on water diffusion properties. The main advantages of tractography are that it allows tracts to be segmented in native space, can account for interindividual differences in structure to a much higher degree than any voxel-wise method and can be performed completely automatically. Furthermore, some algorithms incorporate the uncertainty in the principal diffusion direction at each voxel and can model multiple fiber directions per voxel [34]. Such methods generally give a better representation of the underlying anatomy and allow the tract-averaged FA values to be weighted according to the probability that a voxel is connected to the seed [35].

To segment the genu of corpus callosum, probabilistic neighborhood tractography (PNT) [35], an automatic method which reduces tractography's dependency on seed point location, was applied. First, the seed point of a reference tract derived from a digital human white matter atlas [14] was transferred to each subject's native space. Next, the BedpostX/ProbtrackX tractography algorithm [34] was run with 5000 streamlines and a two-fiber model, for each voxel within a 7×7×7-voxel neighborhood surrounding the seed point, creating a large number of candidate tracts. The PNT algorithm then automatically selects the tract from amongst this group of candidates that best matches the reference tract with respect to shape and length. The segmentations resulting from PNT were visually checked to confirm that none of the tracts were truncated, excessively branched or otherwise deviant from expected anatomy. (An example of a genu segmentation is shown in Supplementary Figure 2.) Average FA values within the segmented tract, weighted according to the likelihood the voxel was connected to the seed, were compared between genotype groups using independent-samples *t* tests for the control group and high-risk group separately. Again, there was one extreme outlier in the high-risk group who was removed in an additional *t* test.

Finally, to verify that any dominant or otherwise nonlinear effects of *ZNF804A* on FA were not obscured by combining the CC and AC genotype groups, we performed analyses of variance of all three genotype groups on average FA within the genu and the corpus callosum SVC.

#### 2.2.7 Power considerations

Post hoc power calculations are controversial because they are often based on the observed effect size involving circular reasoning and a “power paradox” where higher (less significant) *P* values correspond to both lower observed power and more evidence for the null hypothesis [36]. Therefore, we applied more than one methodological approach to our power calculations, none of which were subjected to the circularity problem common to many post hoc power calculations (see Supplementary Materials).

For the ROI and PNT averages, we examined the following: (1) confidence intervals around the observed group differences in relation to effects typically observed in similar studies, (2) the power of our study to detect such a typical effect size and (3) the sample size that would be required to obtain a significant result given our own observed effect size. In each of these calculations, we avoided using both our observed effect size and sample-size-dependent observed statistics at the same time. Details of these power calculations can be found in the Supplementary Methods.

## 3 Results

Allele frequencies of rs1344706 in these samples were similar to those reported previously (Table 1) [1]. There were no significant deviations from Hardy–Weinberg equilibrium. No statistically significant differences (all *P*>.84) between rs1344706 genotype groups were found in age, sex, education and IQ for any of the samples, apart from an age difference in the Scottish control sample (Table 1)*.*

In the German sample, voxel-based analysis of FA, MD or regional brain volumetric measures did not result in any significant differences between rs1344706 genotype groups in any brain region either on the voxel level (all *P*_*FDR*_>.37) or on the cluster level (all *P*>.49; Supplementary Table 1). Similarly, using TBSS, no significant differences in FA were found between the genotype groups in either the control or high-risk samples of the Scottish study (all *P*>.38)*.* No significant differences were found in the Scottish control sample after the model was adjusted for the effect of age (*P*>.37). Histograms of raw *T*-statistics within the TBSS skeletons were symmetrically distributed around zero.

**Fig. 1:**
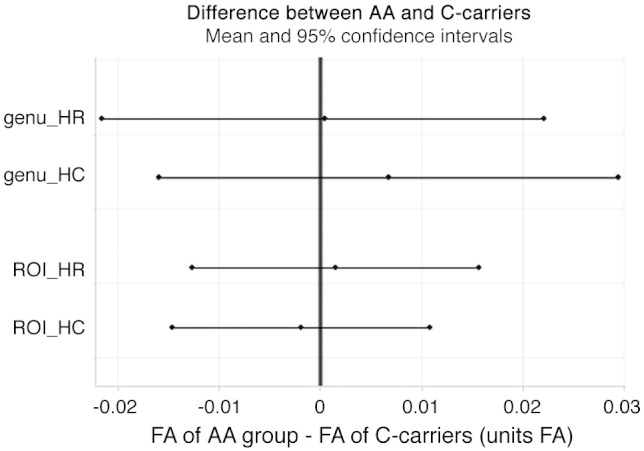
*ZNF804A* effect sizes on FA in the anterior corpus callosum in the Scottish samples. Ninety-five percent confidence intervals around mean genotype group differences in the PNT and ROI analyses in the Scottish samples. HC=healthy control group, HR=high-risk group, ROI=region of interest of genu & body of corpus callosum. Note that typical effect sizes for FA range between 0.05 and 0.10 FA units, which lie comfortably outside the range of the 95% confidence intervals of our comparisons.

The application of an SVC over the body and genu of corpus callosum did not result in any FA differences between *ZNF804A* genotype groups using voxel-wise statistics with TFCE (*P*>.37). Average FA within the corpus callosum ROI also did not differ between genotype groups for the control (*T*=−0.29, *P*=.78) and high-risk (*T*=−0.23, *P*=.82) groups. Correspondingly, no significant genotype effects were found with PNT for genu in the Scottish samples (controls: *T*= 0.58, *P =*.57; high-risk group: *T*= 0.55, *P =*.58). Removal of the extreme outlier in the high-risk group did not change this negative result (tractography: *T*= 0.02, *P*=.99; ROI: *T*= 0.20, *P*=.84). As shown in Fig. 1, there were no trends in either direction for any of the comparisons. Finally, analyses of variance comparing all three genotype groups with respect to average FA within the genu and body of corpus callosum ROI were all nonsignificant, with or without outlier (all *F*< 0.75, all *P*>.49), indicating that there were no nonlinear or dominant effects of the risk allele that may have been obscured by combining the CC and AC groups.

In summary, whole-brain, TBSS, ROI and PNT results were consistently negative. This finding is unlikely to be due to a lack of statistical power since, based on observed effect sizes and the most optimistic of power calculations, a sample size of more than 7000 people would be needed to produce a significant positive finding. Furthermore, other genes previously associated with FA typically showed group differences between 0.05 and 0.10 FA units, which are far outside the 95% confidence intervals based on our results (Fig. 1); this study had 83% power to detect an FA difference as small as 0.02 (see Supplementary Methods for full details).

## 4 Discussion

To date, the rs1344706 locus in *ZNF804A* is statistically the best supported SNP in association with schizophrenia and the wider psychosis phenotype [1–5], but the mechanisms by which it may affect susceptibility to psychosis are poorly understood. Associations of *ZNF804A* with cognitive and imaging phenotypes [19,20,22,23,37–39] indicate that the gene modulates brain function and is involved in higher cognitive processes.

Here, we present a thorough investigation of the relationship between genotype at rs1344706 of the *ZNF804A* gene and white matter integrity of the brain. Our study was motivated by a strong a priori hypothesis based on previous associations of this SNP with task-independent functional connectivity [20,22], the recent knowledge that the risk genotype at this SNP is responsible for creating a myelin transcription factor binding site [2,19] and FA as an established intermediate phenotype [17]. Despite the use of various analyses methods and efforts to increase statistical power in three adequately sized samples, results were remarkably and consistently negative. No trends were observed, with group means differing randomly in either direction and histograms of *T*-statistics normally distributed around zero. Quantitative power calculations all suggested that if there were any true effects, they must be far smaller than what is typical for imaging genetics studies to have remained undetected in the present study. Moreover, such a small effect would only be able to explain a small portion of the strong associations (*z*> 3.5) between *ZNF804A* and prefrontal functional coupling previously reported [16,20–22] and thus would have limited mediating power.

There are several possible explanations for the apparent discrepancy between the effects of *ZNF804A* on task-independent functional connectivity [20,22] and its lack of effect on structural connectivity. With regard to methodology, possible explanations are population heterogeneity, lack of statistical power in the current study and limitations of both DT-MRI- and fMRI-based functional connectivity methods. In general, the interpretation of functional connectivity derived from fMRI is complicated by factors such as task-dependent effects, the possibility of activation in a third area modulating the correlation between two areas of interest, as well as the inherent limitations of fMRI including regional differences in blood-oxygen-level dependence response, and limited temporal and spatial resolution. Hence, the relationship of functional connectivity to structural connectivity is not entirely clear. On the other hand, DT-MRI is also limited by spatial resolution and tensor modeling, and voxel-wise FA analysis is obscured by co-registration errors, partial volume effects and the arbitrary choice of smoothing kernels [40]. TBSS is less susceptible to these nuisance effects, but is limited by nonstationarity (of variance) across the skeleton [41]. However, as we showed consistent results across both of these methods, as well as in the ROI and PNT analyses, the limitations of specific DT-MRI processing pipelines are unlikely to have affected all of our results simultaneously. A more important limitation of DT-MRI here is that the scale at which FA is measured means it would fail to detect small-scale differences in structural integrity, especially when at the synapse or near the gray matter, away from large fiber bundles. It is also possible that the reported effects of *ZNF804A* were sample specific since most previous observations of *ZNF804A* effects on cognitive and imaging phenotypes were derived from the same or largely overlapping samples [20,22,37], and recent replication efforts have not been entirely consistent, with one replication [16] which did not survive multiple testing corrections and another study replicating the frontotemporal connectivity results but not the interhemispheric prefrontal disconnectivity [21].

Perhaps the most likely explanation is that *ZNF804A* has an effect on functional connectivity but not on white matter structure, for example, by interacting with neurotransmitter synthesis or release, with receptor affinity or density, or because of common thalamic input. Gray matter integrity is also a possible mediator, for example, through local dendrite density or growth or, as suggested in Ref. [19], oligodendrocytes within the cortical neuropil. The latter is compatible with the A-allele in rs1344706 creating a myelin transcription factor binding site [2,19] and with the association with regional variation in cortical thickness. In vitro and animal research into the molecular and cellular functions of *ZNF804A* should investigate the plausibility of such mechanisms.

## 5 Conclusion

We were unable to detect any effects of *ZNF804A* genotype on white matter integrity in any of our three samples using four different DT-MRI analysis methods. This is the second [19] thorough investigation, using state-of-the-art imaging methods and adequate sample sizes, reporting no association of *ZNF804A* with FA in healthy individuals. These data therefore suggest that task-independent effects of *ZNF804A* on interhemispheric prefrontal functional connectivity are unlikely to be mediated by structural integrity differences in the corpus callosum.

## Figures and Tables

**Table 1:** Demographic characteristics of the three samples

rs1344706	German sample			Scottish control sample			Scottish high-risk sample		
	AA	AC+CC	*P*	AA	AC+CC	*P*	AA	AC+CC	*P*
*N*	19	24+7		31	39+13		37	36+11	
Age (years)	23.1±1.2	22.5±2	.20	21.8±2.3	20.7±2.4	.03	21.4±2.8	21.4±2.8	.94
Sex (M/F)	9/10	16/15	.78	14/17	23/29	.93	17/20	22/25	.94
IQ	117.9±8	117.3±12.5	.91	111.7±11.9	112.2±13.1	.84	108.0±13.9	104.6±14.4	.28

## Appendix A Supplementary data

SupMat_for_MRI.
