# Supplementary material for: An investigation of a genomewide supported psychosis variant in ZNF804A and white matter integrity in the human brain
Source: Magn Reson Imaging. 2012 Dec;30(10):1373–80. doi: 10.1016/j.mri.2012.05.013 (PMC3778890; doi:10.1016/j.mri.2012.05.013)
Supplement: Supplementary file 1 — SupMat_for_MRI. [file mmc1.pdf]

SUPPLEMENTARY MATERIALS

INDEX:

1. **Power considerations** p. 2
2. **Supplementary References** p. 5
3. **Supplementary Figure 1** p. 6
4. **Supplementary Table 1** p. 7

POWER CONSIDERATIONS

Considering that our sample size is typical for imaging genetics studies, and we have previously found significant effects of genetic variants on FA in these samples [1-3], our samples should be large enough to detect effects typical for MRI. Also, our negative result was repeated in three independent samples, one of which concerned people who are genetically more vulnerable to psychiatric disorder, which theoretically could make effects of specific genes more pronounced. In addition, we applied both whole brain voxel-wise analysis in order not to overlook any effects in unexpected locations, as well as a small volume correction, quantitative tractography and ROI-based methods to increase power in the hypothesised regions. Despite these efforts to we did not detect any significant effects.

In general, when the location of a hypothesized effect is expected *a priori*, ROI analysis should be statistically more powerful than whole brain voxel-wise analyses because it does not require a whole brain correction for multiple comparisons. In this case, to confirm the hypothesis that structural connectivity mediates the effect of *ZNF804A* on inter-hemispheric prefrontal functional connectivity, the location of any effects of *ZNF804A* on FA would be in the anterior part of the corpus callosum, which contains fibers connecting the prefrontal areas in question.

Post-hoc power calculations are a controversial endeavour [4], partly because they often involve a certain amount of circularity in which one or more statistically dependent parameters are estimated from the same analysis that is evaluated. Therefore, the aim here is not simply to perform a traditional power analyses and state the observed power or the desired effect size. Instead we evaluated the following aspects of statistical power: (1) confidence intervals around the observed group differences in relation to effects typically observed in similar studies, (2) the power of our study to detect such an effect size, and (3) the sample size that would be required to obtain a significant result given our own observed effect size. Of note, in all three evaluations we avoided the circularity so common to power analyses by refraining from deriving both the

effect size and sample-size dependent measures, i.e. power, P-value or sample size, from our own analyses.

In three out of the four corpus callosum analyses (quantitative tractography and ROI for each sample), individuals homozygous for the risk-allele (A) had on average higher FA than C-carriers in both high risk and control groups, the opposite of what was hypothesized. Only for the corpus callosum ROI in the healthy controls was the effect of *ZNF804A* genotype in the expected direction. This is illustrated in Figure 2, with 95% confidence intervals around the mean difference between genotype groups.[1] This figure also shows that the maximum effect under the 95% confidence interval in either direction is just below 0.03 FA units. The maximum difference in the expected direction under the 95% confidence interval is 0.0216 FA units, for genu in the high-risk group. In comparison, we previously reported a significant effect of neuregulin-1 on FA in the left anterior thalamic radiation with an effect size of around 0.07 FA units [2], and voxel-based FA studies reported effects of BDNF [5,6], ErbB4 [1] and neuregulin-1 [3] genotypes of 0.05 to 0.11 FA units. These effects sizes are far outside the confidence intervals in the present study and we can therefore be fairly confident that *ZNF804A* has no such effect on FA in the corpus callosum. However, Liu et al. [7] report effects of COMT haplotypes around 0.2 FA units, marginally within the scope of our widest confidence interval only, namely genu in high-risk subjects.

We calculated that with our own sample size and standard deviation we had 83% power to detect an effect of 0.02 FA units, in line with the lowest mean FA difference in similar studies and corresponding to d ~ 0.5; two-tailed T-test with $P < 0.05$.[2] Note that this is not a post-hoc

---

[1] CI plot: http://www.healthstrategy.com/meta/meta.pl

[2] Power calculation: http://www.stat.uiowa.edu/~rlenth/Power/index.html

power analysis, as is disputed in Hoenig et al. [4], because we did not use our observed effect size to calculate it. However, it should be noted that our observed standard deviations are only an estimate of the true standard deviations in the population.

In the ROI analysis of the control group, individuals homozygous for the risk allele (AA) had on average 0.0019 lower FA than C-allele carriers, with a standard deviations of SD_{AA} = 0.02552 and $SD_{C\text{-}car}$ = 0.02875. Using SAS (http://www.sas.com), we computed that for a study to have 80% power to detect a difference of 0.0019 FA units in a two-tailed T-test, given the observed within-group variances and equal genotype groups, a sample size of N = 7324 would be required. We refrained from calculating required sample sizes for the other three analyses because there the AA group had on average higher FA than the C-carriers, opposite of what was hypothesized.

We did not correct for multiple testing in these ROI analyses, because here it is more important to reduce type-II than type-I error. However, it should be borne in mind that if our results were positive, a correction for multiple testing would be required and would increase P-values substantially. Therefore all the above calculations are under-estimations of the true requirements to obtain significance after correction for multiple testing.

References

1. Konrad A, Vucurevic G, Musso F, Stoeter P, Dahmen N, Winterer G. ErbB4 genotype predicts left frontotemporal structural connectivity in human brain. Neuropsychopharmacology 2009;34(3):641-650.

2. Sprooten E, Lymer GKS, Muñoz Maniega S, McKirdy J, Clayden JD, Bastin ME, et al. The relationship of anterior thalamic radiation integrity to psychosis risk associated neuregulin-1 variants. Mol Psychiatry 2009;14(3):237-238, 233.

3. Winterer G, Konrad A, Vucurevic G, Musso F, Stoeter P, Dahmen N. Association of 5' end neuregulin-1 (NRG1) gene variation with subcortical medial frontal microstructure in humans. NeuroImage 2008;40(2):712-718.

4. Hoenig JM, Heisey DM. The Abuse of Power. The American Statistician 2001;55(1):19-24.

5. Chiang M-C, Avedissian C, Barysheva M, Toga AW, McMahon KL, de Zubicaray GI, et al. Extending genetic linkage analysis to diffusion tensor images to map single gene effects on brain fiber architecture. Med Image Comput Comput Assist Interv 2009;12(Pt 2):506-513.

6. Chiang M-C, Barysheva M, Toga AW, Medland SE, Hansell NK, James MR, et al. BDNF gene effects on brain circuitry replicated in 455 twins. NeuroImage 2011;55(2):448-454.

7. Liu, B, Li, J, Yu, C, Li, Y, Liu, Y, Song, M, et al. Haplotypes of catechol-O-methyltransferase modulate intelligence-related brain white matter integrity. NeuroImage 2010;50(1):243-249.

SUPPLEMENTARY FIGURE 1

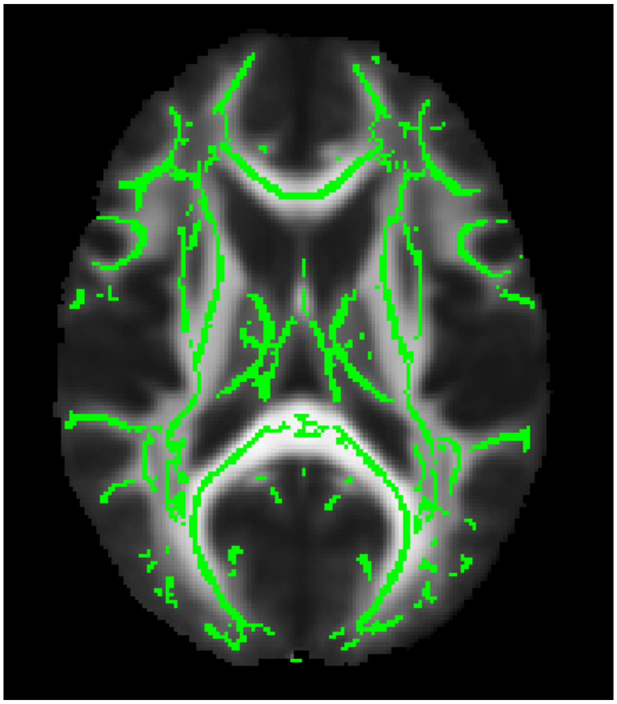

TBSS white matter skeleton template overlaid on the mean FA volume.

SUPPLEMENTARY FIGURE 2

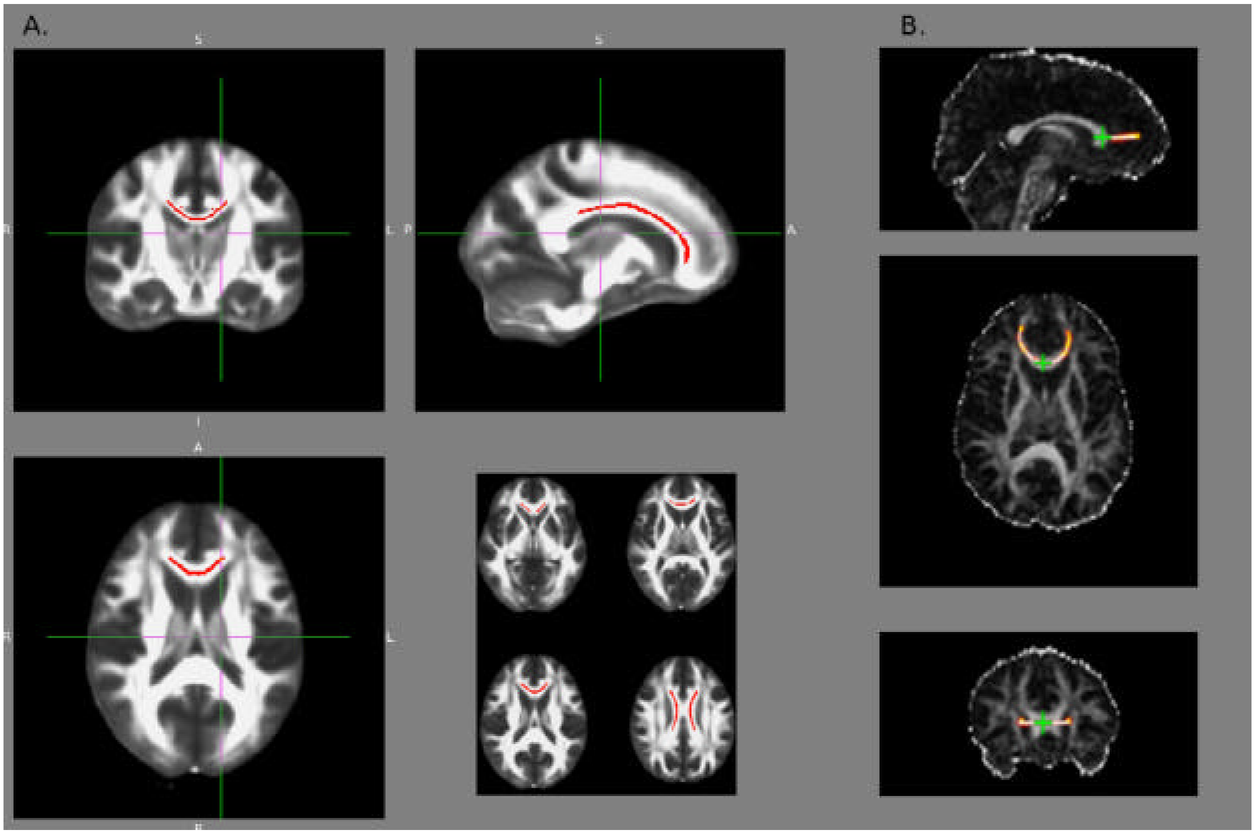

A: Creation of the small volume correction mask of the genu and body of corpus callosum, which was also used for the ROI analysis. **B:** Typical example of genu segmentation resulting from PNT. The yellow-red scale represents the number of streamlines passing through a voxel, and the green cross indicates the location of the seed point.

SUPPLEMENTARY TABLE 1

Clusters (> 20 voxels) with the most prominent differences of fractional anisotropy (FA) and mean diffusivity (MD) between genotype groups, thresholded at a *P* < 0.001, uncorrected.

**DTI measure**	**Contrast**	**Brain region**	**Peak** (MNI)	**Peak T**	**P – value*** (cluster level)	**Cluster size** (voxels)
FA	AA > AC + CC	R internal capsule	-26, -4, 16	3.70	0.892	25
		R superior longitudinal fasciculus	-22, -4, 38	3.70	0.849	55
		R middle temporal gyrus	-52, -48, 8	3.68	0.736	31
		R inferior longitudinal fasciculus	-40, -24, -6	3.79	0.752	31
MD	AA < AC + CC	L rectus gyrus	10, 42, -14	3.83	0.494	73
		R anterior corona radiata	-24, 42, 12	3.67	0.776	46

L = left; R = right
MNI = Montreal Neurological Institute
*Cluster-wise corrected for whole brain volume using non-stationary cluster inference toolbox
